# Comparative analysis of progression milestones across Parkinson’s disease clinical, pathological, and data-driven subtypes: a 10-year follow-up

**DOI:** 10.1038/s41531-026-01430-8

**Published:** 2026-06-12

**Authors:** Ahmed Negida, Nitai Mukhopadhyay, Brian D. Berman, Matthew J. Barrett

**Affiliations:** 1 Department of Neurology, Virginia Commonwealth University, Richmond, VA USA; 2 Department of Biostatistics, Virginia Commonwealth University, Richmond, VA USA

**Keywords:** Biomarkers, Diseases, Medical research, Neurology

## Abstract

Parkinson’s disease (PD) heterogeneity complicates disease-modification clinical trial design and lead to ambiguous results, necessitating robust subtyping frameworks to identify rapid progressors. This study compared progression milestones across previously defined clinical (tremor-dominant [TD], postural instability/gait difficulty [PIGD], indeterminate), pathological (brain-first, body-first), and data-driven (diffuse malignant [DM], intermediate [IM], mild motor-predominant [MMP]) PD subtypes. Using data from the Parkinson’s Progression Markers Initiative (PPMI), we analyzed milestone attainment across six functional domains and performed Cox proportional hazards regression. Randomized controlled trial sample size simulations evaluated the impact of subtyping on trial efficiency. Data-driven subtypes exhibited the highest progression rates, with DM patients attaining 63.0% of milestones, surpassing PIGD (55.6%) and body-first (54.0%) subtypes. Cox proportional hazards regression confirmed that DM patients had the highest hazard of progression compared to MMP, after adjusting for age at enrollment. Trial power simulations demonstrated that enrolling DM patients could reduce sample size requirements by approximately 50% using standard trial durations compared to unstratified PD cohorts. This analysis showed that data-driven subtyping, particularly the identification of the DM subtype, offers a promising strategy to optimize disease-modification trials in PD by capturing patients who meet progression milestones earlier.

## Introduction

Parkinson’s disease (PD) is a progressive neurodegenerative disorder characterized by the accumulation of misfolded α-synuclein aggregates and the selective vulnerability of dopaminergic, cholinergic, and noradrenergic neuronal populations^1–6^. Beyond the classical motor triad, PD encompasses a broad spectrum of non-motor manifestations—including REM sleep behavior disorder, autonomic dysfunction, hyposmia, and psychiatric symptoms, some of which may precede motor onset by decades^7–9^. This clinical complexity gives rise to marked heterogeneity in symptom profiles and disease progression trajectories.

Current therapies, primarily focused on dopamine replacement (e.g., levodopa), provide symptomatic relief for motor symptoms but fail to modify disease progression^10,11^. Currently, there are no available pharmacological therapies that can stop or slow the disease progression, despite the several disease-modification trials that have been conducted over the last three decades. Two major challenges are believed to contribute to the failure of disease-modifying trials in PD to date: (1) the substantial heterogeneity in pathology, symptoms, and progression trajectories, and (2) the incremental progression of the disease in most patients, requiring long-term follow-up to demonstrate disease-modifying benefits. Together, these challenges undermine conventional trial designs, which typically rely on short-term follow-up periods (1–2 years) insufficient to capture clinically meaningful progression in many patients.

To address the heterogeneity seen in PD, efforts have been made to stratify PD patients into biologically or clinically homogeneous subtypes^12–16^. The most widely described PD subtypes include (1) clinical classifications, such as tremor-dominant (TD) and postural instability/gait difficulty (PIGD)^17,18^; (2) pathological models, including brain-first and body-first subtypes, which hypothesize distinct etiological pathways^16,19^; and (3) data-driven categories, such as the diffuse malignant (DM), intermediate (IM), and mild motor-predominant (MMP) subtypes, identified through machine learning-based cluster analysis^14,15^.

Despite these efforts, critical limitations hinder the translation of subtyping research into improved clinical trial design. The Movement Disorders Society Task Force on the definition of PD stated that “*subtypes should only be delineated if there are clear data that demonstrate consistent, large differences in prognosis, predicted disease manifestations, or treatment*.”^20^ and the literature emphasized that the key criteria for PD subtyping are biomarker profile and longitudinal progression^14,15^. Existing subtyping models have not been directly compared for their prognostic accuracy and most studies rely on cross-sectional data without demonstrating, through longitudinal study, the prognostic value of these subtypes in future clinical trials of potential disease modifying interventions. This knowledge gap limits the applicability of PD subtypes in optimizing disease-modification clinical trials, where enrolling rapid progressors is important to detect treatment effects efficiently. Our study addresses this gap by answering an important research question “*Which existing PD subtype classification—clinical, pathological, or data-driven—most effectively identifies individuals with the fastest progression across six key domains: motor complications, gait/balance, cognition, autonomic dysfunction, functional dependence, and activities of daily living?*”. Using longitudinal data from the Michael J. Fox Foundation’s Parkinson’s Progression Markers Initiative (PPMI) cohort, we tracked 25 disease progression milestones for each of the 3 subtyping models and simulated clinical trials to assess the utility of each subtyping model. The goal of this work was to identify subtypes that enhance trial sensitivity to detect treatment effects, thereby accelerating therapeutic development.

## Results

### Clinical characteristics of the study groups

A total of 1,418 patients from the PPMI cohort met inclusion criteria. Table 1 summarizes the baseline characteristics across clinical, pathological, and data-driven subtypes. Age at enrollment, age at symptom onset, and age at diagnosis did not significantly differ across clinical subtypes (TD, PIGD, Indeterminate; *P* = 0.174, *P* = 0.479, *P* = 0.373, respectively) or between pathological subtypes (Brain-first vs Body-first; *P* = 0.117, *P* = 0.331, *P* = 0.197, respectively). However, body-first patients had a higher proportion of males (69.5% vs 61.2%, *P* = 0.006). Data-driven subtypes showed significant differences in age at enrollment, symptom onset, and diagnosis (all *P* < 0.001), with DM patients being the oldest (median enrollment age 66.6 years) compared to IM (65.2) and MMP (61.8). Male sex distribution was comparable across clinical (*P* = 0.340) and data-driven (*P* = 0.183) subtype groups.

**Table 1:** Baseline clinical characteristics across Parkinson’s disease subtypes

Clinical subtypes					
	*N*	TD (*n* = 911)	PIGD (*n* = 374)	Indeterminate (*n* = 133)	*P* value
Age at enrollment	1418	64.1 (56.7–70.1)	63.5 (56.0–69.8)	63.3 (53.7–69.4)	0.174
Age at symptom onset	1402	61.2 (53.8–67.4)	60.8 (53.1–67.5)	60.0 (51.5–66.9)	0.479
Age at diagnosis	1418	62.8 (55.5–68.9)	62.2 (54.9–68.9)	62.4 (53.2–67.9)	0.373
Sex: male	1418	575/911 (63.1%)	220/374 (58.8%)	84/133 (63.2%)	0.340
MDS-UPDRS I	1408	1.0 (0.0–2.0)	1.0 (0.0–3.0)	1.0 (0.0–2.0)	<0.001*
MDS-UPDRS II	1415	5.0 (2.0–8.0)	8.0 (5.0–12.0)	6.0 (3.0–9.0)	<0.001*
MDS-UPDRS III (off)	1334	21.0 (16.0–29.0)	21.0 (14.0–29.0)	19.0 (14.0–26.0)	0.015*
MDS-UPDRS total	1324	28.0 (21.0–37.0)	32.0 (22.0–42.0)	25.0 (19.0–35.0)	<0.001*
UPSIT total	688	23.0 (18.0–29.0)	22.0 (16.0–29.0)	24.0 (15.0–30.0)	0.799
MoCA total	1413	27.0 (25.0–29.0)	27.0 (25.0–28.0)	28.0 (26.0–29.0)	0.175
RBDSQ total	1418	3.0 (2.0–5.0)	4.0 (2.0–7.0)	3.0 (2.0–6.0)	<0.001*
SCOPA-AUT total	1410	9.0 (5.0–13.0)	11.0 (7.0–16.0)	10.0 (7.0–15.0)	<0.001*

Pathological subtypes					
	*N*	Brain-first (*n* = 747)	Body-first (*n* = 374)		*P* value
Age at enrollment	1121	63.5 (56.1–69.8)	64.8 (57.3–69.9)		0.117
Age at symptom onset	1110	60.9 (53.4–67.2)	61.8 (53.6–67.7)		0.331
Age at diagnosis	1121	62.2 (55.0–68.8)	63.8 (55.6–69.0)		0.197
Sex: male	1121	457/747 (61.2%)	260/374 (69.5%)		0.006*
MDS-UPDRS I	1112	1.0 (0.0–2.0)	1.0 (0.0–3.0)		<0.001*
MDS-UPDRS II	1119	5.0 (2.0–8.0)	7.0 (5.0–12.0)		<0.001*
MDS-UPDRS III (off)	1052	20.0 (15.0–27.0)	22.0 (16.0–31.0)		0.004*
MDS-UPDRS total	1043	27.0 (20.0–35.0)	33.0 (23.0–43.0)		<0.001*
UPSIT total	564	24.0 (18.0–29.0)	21.0 (15.0–28.0)		0.008*
MoCA total	1116	27.0 (25.0–29.0)	27.0 (25.0–28.0)		0.038*
RBDSQ total	1121	2.0 (1.0-3.0)	8.0 (7.0–9.0)		<0.001*
SCOPA-AUT total	1114	8.0 (5.0–12.0)	12.0 (8.0–18.0)		<0.001*

Data-drivensubtypes					
	*N*	MMP (*n* = 600)	IM (*n* = 501)	DM (*n* = 219)	*P* value
Age at enrollment	1320	61.8 (54.8–67.9)	65.2 (56.7–70.2)	66.6 (59.3–72.3)	*P* < 0.001*
Age at symptom onset	1308	59.8 (52.4–65.1)	62.5 (54.3–68.2)	63.8 (55.6–69.7)	*P* < 0.001*
Age at diagnosis	1320	61.1 (54.0–67.0)	64.1 (55.9–69.4)	65.2 (57.5–71.0)	*P* < 0.001*
Sex: male	1320	362/600 (60.3%)	324/501 (64.7%)	145/219 (66.2%)	0.183
MDS-UPDRS I	1312	1.0 (0.0–2.0)	1.0 (0.0–3.0)	2.0 (1.0–4.0)	*P* < 0.001*
MDS-UPDRS II	1320	4.0 (2.0–6.0)	6.0 (4.0–9.0)	12.0 (10.0–15.0)	*P* < 0.001*
MDS-UPDRS III (off)	1320	18.5 (14.0–24.0)	21.0 (16.0–28.0)	32.0 (24.0–39.0)	*P* < 0.001*
MDS-UPDRS total	1312	24.0 (18.0–31.0)	29.0 (22.0–38.0)	46.0 (39.0–56.0)	*P* < 0.001*
UPSIT total	658	24.0 (18.0–29.0)	22.0 (15.0–28.0)	22.5 (16.0–28.0)	*P* = 0.019*
MoCA total	1320	28.0 (27.0–29.0)	27.0 (24.0–28.0)	26.0 (23.0–28.0)	*P* < 0.001*
RBDSQ total	1320	2.0 (1.0–4.0)	4.0 (2.0–7.0)	7.0 (3.0–9.0)	*P* < 0.001*
SCOPA-AUT total	1320	7.0 (4.0–10.0)	11.0 (7.0–15.0)	17.0 (12.0–22.0)	*P* < 0.001*

Values are presented as median (interquartile range) unless otherwise indicated. N is the number of non-missing values. P-values are based on the Kruskal-Wallis test for continuous variables compared across 3 subtypes (clinical and data-driven subtypes), Wilcoxon rank-sum test for continuous variables compared across 2 subtypes (pathological subtypes), and Pearson chi-square test for categorical variables.

Motor severity differed across all subtype classifications. PIGD patients exhibited higher baseline MDS-UPDRS II scores compared to TD (median 8.0 vs 5.0, *P* < 0.001), with similar patterns for MDS-UPDRS total scores (32.0 vs 28.0, *P* < 0.001). Body-first patients had higher MDS-UPDRS II and III scores than brain-first patients (median 7.0 vs 5.0 and 22.0 vs 20.0; both *P* < 0.01). Among data-driven subtypes, DM patients demonstrated the highest baseline motor burden (MDS-UPDRS II: median 12.0; MDS-UPDRS III: median 32.0; total: median 46.0; all *P* < 0.001). Regarding cognitive function, no significant differences in baseline MoCA scores were observed across clinical subtypes (*P* = 0.175). However, body-first patients had marginally lower MoCA scores than brain-first patients (both median 27.0, *P* = 0.038). Among data-driven subtypes, DM patients had significantly lower MoCA scores (median 26.0) compared to IM (27.0) and MMP (28.0; *P* < 0.001), consistent with cognition being a defining feature of this subtyping framework. SCOPA-AUT scores were highest in DM patients (median 17.0 vs 11.0 for IM and 7.0 for MMP; *P* < 0.001), reflecting greater baseline autonomic dysfunction.

### Biomarkers in different PD subtypes

No significant differences in CSF biomarker levels (Aβ42, pTau, tTau, alpha-synuclein, or NfL) were observed across clinical or pathological subtypes. No significant differences were found in CSF Aβ₄₂ or NfL concentrations across clinical or pathological subtypes. However, data-driven subtypes showed significant differences in NfL levels (Table 2). In the pairwise group comparisons following significant omnibus tests, serum NfL concentrations were significantly elevated in the DM subtype compared to the MMP and IM subtypes (*P* < 0.01).

**Table 2:** CSF and serum biomarker levels across Parkinson’s disease subtypes

Clinical subtypes					
	*N*	TD (*n* = 352)	PIGD (*n* = 167)	Indeterminate (*n* = 59)	*P* value
CSF Aβ 1-42 (pg/mL)	480	836.3 (613.0–1125.0)	811.6 (613.5–1072.5)	841.7 (585.4–1007.0)	0.92
CSF pTau (pg/mL)	547	13.4 (10.9–17.2)	13.4 (11.0–17.7)	12.2 (10.4–17.8)	0.737
CSF tTau (pg/mL)	578	155.4 (124.1–202.7)	157.0 (124.2–200.6)	148.4 (124.1–202.4)	0.899
CSF α-syn (pg/mL)	485	1375.0 (1055.7–1810.8)	1383.4 (942.4–1784.5)	1407.9 (1076.0–1706.7)	0.713
CSF NfL (pg/mL)	217	89.1 (67.0–120.1)	81.2 (57.5–115.3)	100.8 (74.3–117.0)	0.309
Serum NfL (pg/mL)	569	11.9 (8.7–16.9)	12.1 (9.1–16.6)	10.9 (7.8–15.2)	0.316

Pathological subtypes				
	*N*	Brain-first (*n* = 279)	Body-first (*n* = 153)	*P* value
CSF Aβ 1-42 (pg/mL)	361	812.9 (644.0–1043.0)	847.8 (582.4–1156.0)	0.796
CSF pTau (pg/mL)	412	12.9 (10.7–16.8)	14.7 (11.0–18.3)	0.05
CSF tTau (pg/mL)	432	153.0 (124.1–196.6)	167.5 (122.9–213.3)	0.093
CSF α-syn (pg/mL)	363	1367.7 (1060.7–1759.5)	1375.5 (935.5–1831.0)	0.716
CSF NfL (pg/mL)	160	82.2 (63.8–117.0)	95.1 (68.0–118.3)	0.253
Serum NfL (pg/mL)	429	11.9 (8.6–16.9)	11.6 (8.6–16.8)	0.838

Data-driven subtypes					
	*N*	MMP (*n* = 252)	IM (*n* = 207)	DM (*n* = 91)	*P* value
CSF Aβ 1-42 (pg/mL)	464	803.8 (610.5–1051.0)	841.1 (622.5–1106.0)	910.0 (615.5–1128.0)	0.54
CSF pTau (pg/mL)	520	12.7 (10.6–16.8)	13.7 (10.9–17.5)	14.3 (11.8–17.7)	0.087
CSF tTau (pg/mL)	550	149.1 (123.8–198.6)	157.3 (122.5–204.3)	164.9 (139.6–205.7)	0.267
CSF α-syn (pg/mL)	468	1375.5 (1034.7–1759.5)	1432.7 (1085.0–1795.9)	1374.5 (1032.5–1786.8)	0.815
CSF NfL (pg/mL)	216	78.9 (59.2–108.7)	94.8 (70.3–130.7)	104.2 (65.8–145.2)	0.005
Serum NfL (pg/mL)	534	11.2 (7.9–15.2)	11.8 (8.7–16.9)	14.1 (10.1–19.3)	<0.001

Values are presented as median (interquartile range). N is the number of non-missing values. P-values are based on the Kruskal-Wallis test for comparisons across 3 subtypes (clinical and data-driven subtypes) and Wilcoxon rank-sum test for comparisons of 2 subtypes (pathological subtypes).

*CSF Aβ42* cerebrospinal fluid amyloid-beta 42, *CSF pTau* cerebrospinal fluid phosphorylated tau, *CSF* *tTau* cerebrospinal fluid total tau, *CSF* *α-synuclein* cerebrospinal fluid alpha-synuclein, *CSF* *NfL* cerebrospinal fluid neurofilament light chain, *Serum* *NfL* serum neurofilament light chain.

### Milestone attainment across subtypes

**Fig. 1:**
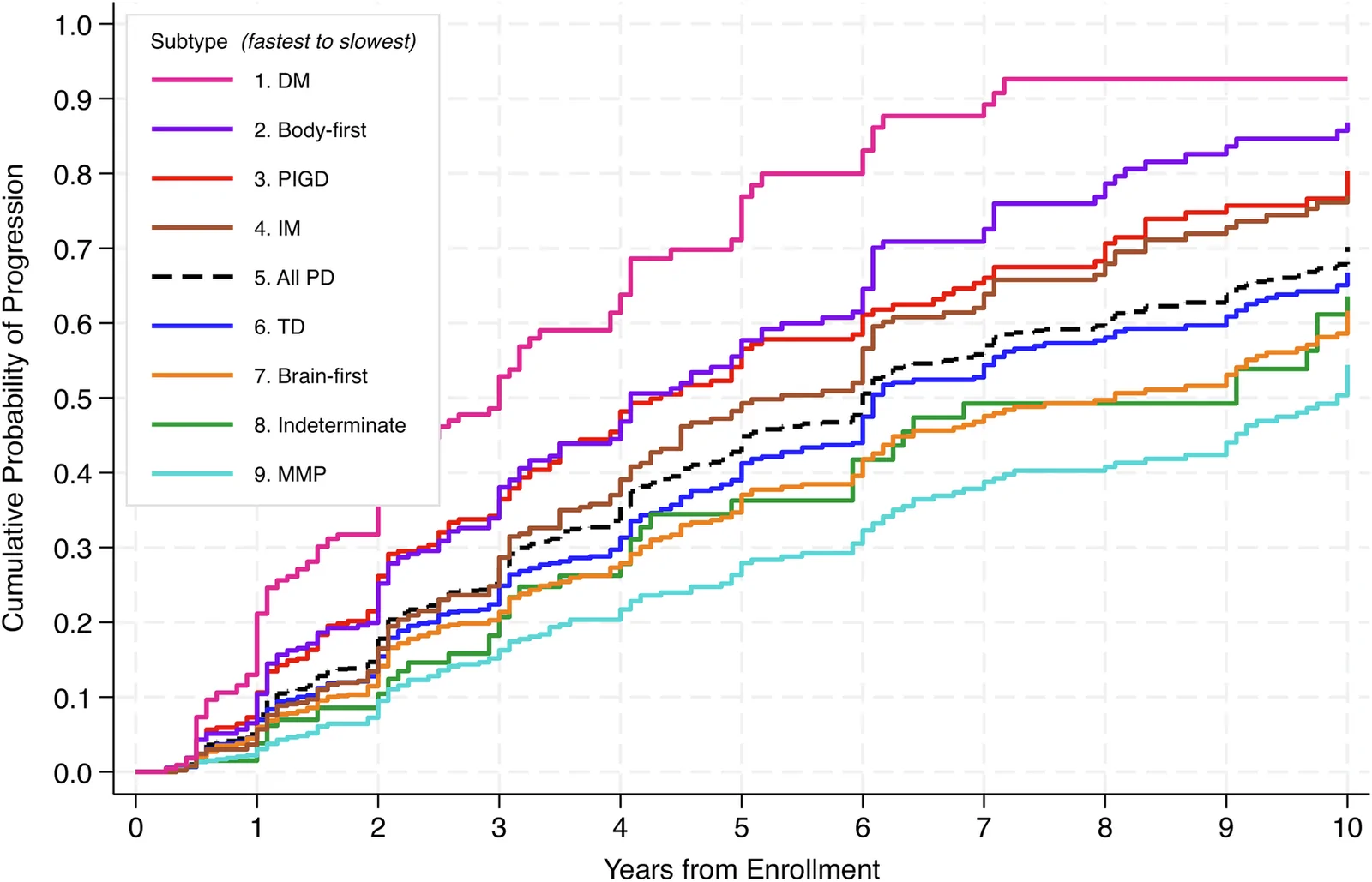
Kaplan–Meier failure functions (1 − survival) showing the cumulative probability of reaching at least one of 25 progression milestones across nine Parkinson’s disease subtypes over 10 years of follow-up. Subtypes are ranked from fastest to slowest progression: DM (data-driven), Body-first (pathological), PIGD (clinical), IM (data-driven), All PD, TD (clinical), Brain-first (pathological), Indeterminate (clinical), and MMP (data-driven). Follow-up was administratively censored at 10 years.

Progression milestone attainment rates over 10 years varied substantially across clinical, pathological, and data-driven subtypes. Among clinical subtypes, the PIGD group showed the highest overall milestone attainment (55.6%), followed closely by the indeterminate subtype (39.1%), and then the TD group (42.7%). For pathological classifications, body-first patients reached more milestones (54.0%) than brain-first patients (39.4%). Notably, among data-driven subtypes, the DM group exhibited the highest rate of progression (63.0%), exceeding the IM (48.9%), the MMP (34.2%), and all other subtypes (Table 3, Fig. 1).

**Table 3:** Percentage of patients reaching progression milestones stratified across the clinical, pathological, and data-driven Parkinson’s Disease Subtypes

Domain	Clinical subtypes			Pathological subtypes		Data-driven subtypes		
	TD (*n* = 911)	PIGD (*n* = 374)	Indeterminate (*n* = 133)	Brain-first (*n* = 747)	Body-first (*n* = 374)	MMP (*n* = 600)	IM (*n* = 501)	DM (*n* = 219)
All milestones	42.7%	55.6%	39.1%	39.4%	54.0%	34.2%	48.9%	**63.0%**
Domain 1: Walking and balance	4.7%	11.2%	5.3%	3.1%	11.8%	3.0%	5.2%	**16.0%**
Domain 2: Motor complications	9.1%	11.5%	7.5%	6.8%	13.4%	7.2%	9.4%	**14.6%**
Domain 3: Cognition	13.3%	18.2%	12.0%	11.2%	20.6%	5.5%	16.6%	**29.7%**
Domain 4: Autonomic dysfunction	14.1%	15.5%	14.3%	9.9%	22.7%	7.5%	17.6%	**23.7%**
Domain 5: Functional dependence	35.1%	46.8%	33.8%	32.1%	45.7%	29.2%	38.9%	**53.4%**
Domain 6: Activities of daily living	2.9%	5.3%	1.5%	2.0%	6.4%	1.5%	2.2%	**10.0%**

Values represent the percentage of patients who ever reached at least one milestone within each domain during follow-up. N is the number of patients in each subgroup. Brain-first and Body-first refer to pathological subtypes.

*TD* Tremor Dominant, *PIGD* Postural Instability Gait Disorder, *MMP* Mild Motor Predominant, *IM* Intermediate, *DM* Diffuse Malignant.

**Fig. 2:**
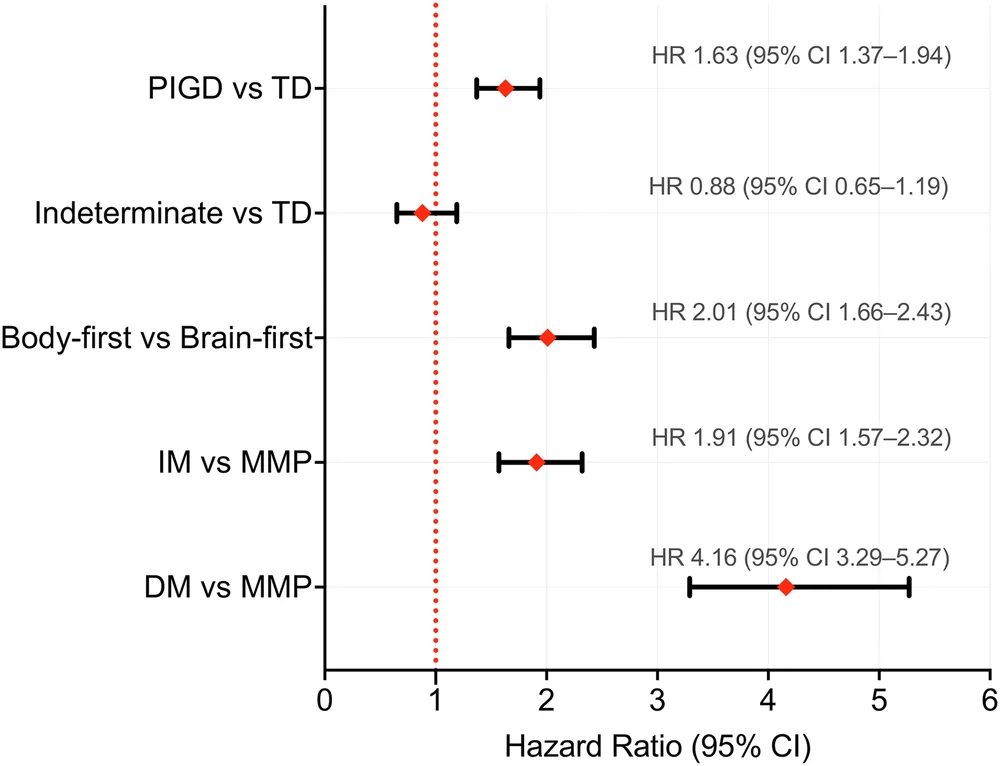
Forest plot summarizing Hazard ratios (HR) and 95% confidence intervals from Cox proportional hazards regression using the Efron method for tied events. Comparisons include clinical (PIGD vs. TD, Indeterminate vs. TD), pathological (Body-first vs. Brain-first), and data-driven subtypes (DM vs. MMP, IM vs. MMP). Reference groups: TD (clinical), Brain-first (pathological), MMP (data-driven). Red dashed line represents HR = 1.0 (no difference). All hazard ratios are from Cox proportional hazards models adjusted for age at enrollment. HR > 1.0 indicates faster progression relative to the reference group. TD Tremor Dominant, PIGD Postural Instability Gait Disorder, MMP Mild Motor Predominant, IM Intermediate, DM Diffuse Malignant.

After excluding patients who had already reached milestones at baseline, similar trends persisted (Table 4, Fig. 2). The DM subtype continued to show the highest incidence of new milestone acquisition (55.0%), followed by PIGD (50.0%) and body-first (47.4%) subtypes. Again, domain-specific patterns emphasized the aggressiveness of the DM subtype, with leading rates in cognition (22.2%) and functional dependence (47.2%).

**Table 4:** Percentage of patients reaching new progression milestones stratified across the clinical, pathological, and data-driven Parkinson’s Disease Subtypes (excluding patients who met milestones at baseline)

Domain	Clinical subtypes			Pathological subtypes		Data-driven subtypes		
	TD (*n* = 847)	PIGD (*n* = 332)	Indeterminate (*n* = 125)	Brain-first (*n* = 706)	Body-first (*n* = 327)	MMP (*n* = 591)	IM (*n* = 455)	DM (*n* = 180)
All milestones	38.4%	50.0%	35.2%	35.8%	47.4%	33.2%	43.7%	**55.0%**
Domain 1: Walking and balance	4.1%	9.3%	5.6%	2.8%	10.1%	3.0%	5.1%	**13.9%**
Domain 2: Motor complications	8.1%	11.1%	8.0%	6.5%	12.2%	7.1%	9.2%	**13.9%**
Domain 3: Cognition	10.5%	14.2%	8.8%	8.9%	15.6%	5.6%	13.4%	**22.2%**
Domain 4: Autonomic dysfunction	12.8%	13.0%	12.8%	9.3%	**19.9%**	7.4%	16.9%	19.4%
Domain 5: Functional dependence	32.1%	41.3%	31.2%	30.0%	39.4%	28.3%	35.4%	**47.2%**
Domain 6: Activities of daily living	2.5%	3.3%	1.5%	1.6%	4.9%	1.5%	2.2%	**6.7%**

Values represent the percentage of patients who reached at least one milestone within each domain during follow-up, after excluding patients who already met the milestone at baseline. N is the number of patients in each subgroup after exclusion. Brain-first and Body-first refer to pathological subtypes.

*TD* Tremor Dominant, *PIGD* Postural Instability Gait Disorder, *MMP* Mild Motor Predominant, *IM* Intermediate, *DM* Diffuse Malignant.

### Survival analysis of disease progression milestones

Progression risk over time differed markedly by subtype, as visualized in Fig. 1. The DM subtype demonstrated the steepest trajectory, with a median time to progression of 3.0 years and an incidence rate of 0.25 events per person-year—nearly four-fold higher than the slowest-progressing subtype, MMP (0.07 events per person-year; median 9.9 years). Body-first and PIGD subtypes showed similarly rapid progression (median 4.1 and 4.3 years, respectively), while TD and Brain-first subtypes progressed more slowly (median 6.1 and 8.1 years, respectively). The overall unstratified PD cohort had a median time to progression of 6.0 years. Notably, 25% of DM patients reached a progression milestone within 1.2 years of enrollment, compared to 4.8 years for MMP, underscoring the potential for subtype-based enrichment in clinical trial design.

These trends were further quantified using the Cox proportional hazard regression model (Fig. 2). Among pathological subtypes, body-first patients had a significantly higher hazard of milestone attainment than brain-first patients (HR = 2.01; 95% CI, 1.66 to 2.43; *P* < 0.001). Within data-driven subtypes, DM patients progressed more than twice as fast as MMP patients (HR = 4.16; 95% CI, 3.29 to 5.27; *P* < 0.001), and IM patients also had elevated risk (HR = 1.91; 95% CI, 1.57 to 2.32; *P* < 0.001). In contrast, differences among clinical subtypes were smaller with marginal statistical significance. Across all models, enrollment age had a modest effect on progression, while subtype classification remained the strongest independent predictor.

### Power-based sample size estimations

**Fig. 3:**
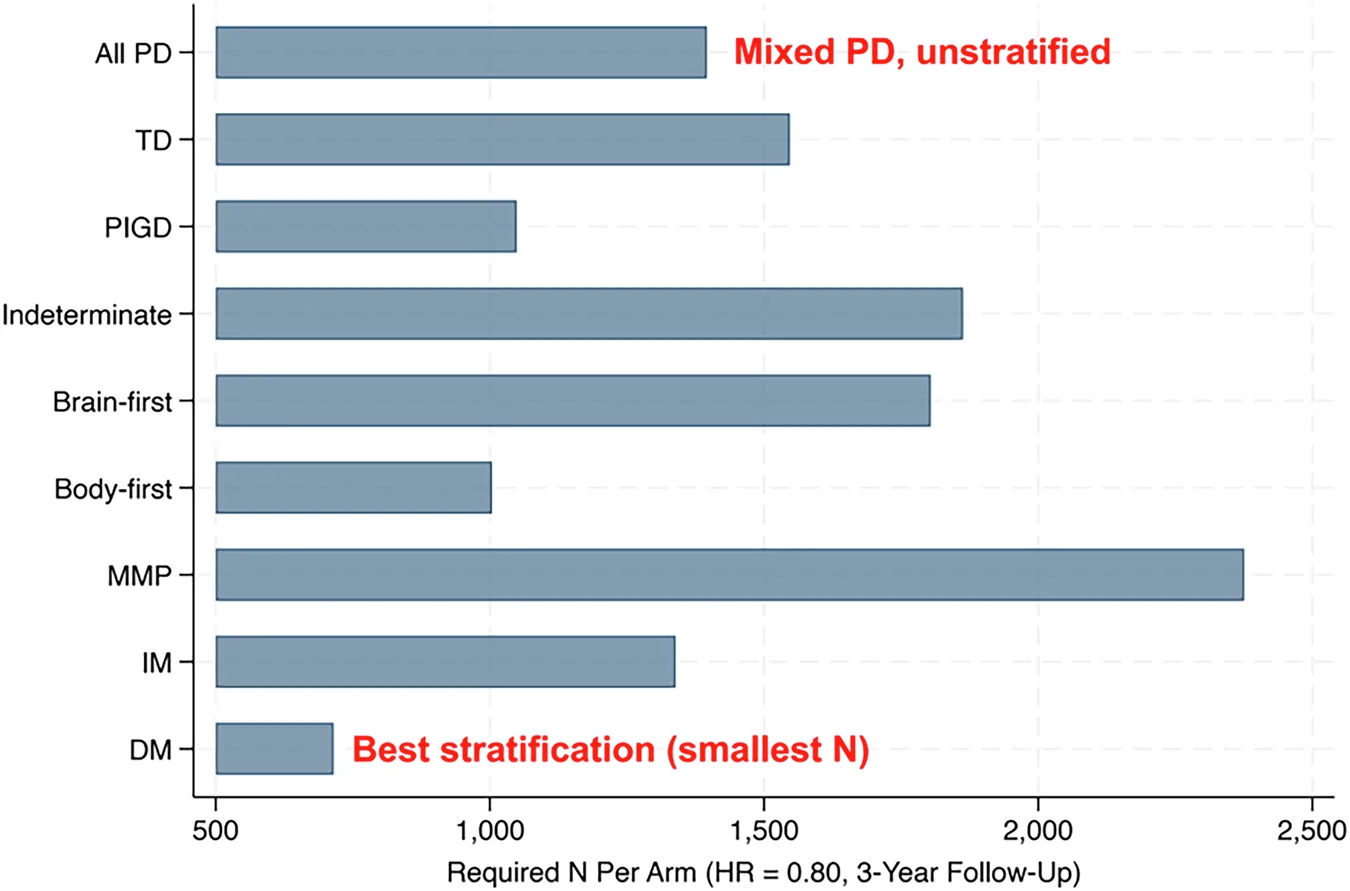
Trial Enrichment Efficiency: N per arm at 3 years; DM-PD = Diffuse Malignant Parkinson’s Disease; Estimated sample size and trial duration requirements for detecting a 20% reduction in progression milestones (HR = 0.80) with 80% statistical power and a two-sided alpha of 0.05. DM subtype trials consistently required smaller sample sizes and shorter durations compared to trials enrolling unstratified PD patients.

We performed sample size estimations using 1 – Kaplan-Meier survival estimates to model cumulative event probabilities. We explored how patient stratification influences trial design efficiency. Assuming a hypothetical disease-modifying therapy that reduces the hazard of reaching a new progression milestone by 20% (HR = 0.80), we estimated the minimum required sample sizes per trial arm to achieve 80% power at a two-sided alpha of 0.05, assuming 10% dropout and 1:1 allocation, using the Schoenfeld (1983) formula. Event probabilities were derived from time-to-event data for each subgroup, with PPMI natural history serving as a proxy for the control arm. As illustrated in Fig. 3, trials enriched for rapidly progressing subtypes required substantially fewer participants than unstratified trials. At a 3-year follow-up, an unstratified PD trial would require 1,396 participants per arm, whereas enrichment for the DM subtype reduced this to 715 per arm (49% reduction). Similarly, enrichment for the PIGD and body-first subtypes reduced requirements to 1049 (25% reduction) and 1004 (28% reduction) per arm, respectively. Conversely, trials restricted to slowly progressing subtypes—MMP (2376/arm) or brain-first (1804/arm)—required substantially larger samples than the unstratified cohort. These findings were consistent across follow-up durations of 1 to 5 years and when assuming other effect sizes (i.e. 30% risk reduction, HR = 0.70), where a 3-year DM-enriched trial required only 293 per arm compared to 576 in the unstratified cohort (Supplementary File 1).

## Discussion

This study showed that data-driven subtyping, particularly the DM subtype, most effectively identifies individuals with PD at highest risk for rapid progression. The DM subtype presents with greater baseline motor and non-motor disease burden compared to other subtype classifications. Further, it exhibited a 63.0% milestone attainment rate over follow-up, significantly outpacing both traditional clinical classifications (TD 42.7%, PIGD 55.6%, Indeterminate 39.1%) and pathological subtypes (Brain-first 39.4%, Body-first 54.0%). Interestingly, DM patients showed accelerated decline across multiple domains, including cognition, motor complications, and functional dependence, with Cox proportional hazards regression confirming a significantly elevated hazard ratio compared to theMMP subtype. In contrast, clinical subtyping offered limited prognostic value, as differences between TD, PIGD, and indeterminate groups were modest and often non-significant. This aligns with prior work by von Coelln et al.^21^, who demonstrated that motor-based subtypes lack temporal stability and exhibit poor reproducibility across timepoints, limiting their use in longitudinal studies and clinical trials. Similarly, while body-first pathological subtypes progressed faster than brain-first, their predictive utility was less than the data-driven subtypes. In this study, no significant differences in CSF biomarker levels (Aβ42, pTau, tTau, alpha-synuclein, or NfL) were observed across clinical or pathological subtypes. However, among data-driven subtypes, serum NfL concentrations were significantly elevated in DM patients compared to MMP and IM (*P* < 0.001), suggesting that peripheral neurofilament release may track with this subtype’s more aggressive phenotype. However, a previous study in China and an earlier analysis of 63 PD patients from PPMI both found significantly higher CSF p-tau181 in the PIGD subtype compared to the TD, which is consistent with our findings^22,23^.

Although non-specific, NfL has emerged as a consistent biomarker of axonal neurodegeneration and poor prognosis in PD. Multiple studies have demonstrated that elevated NfL levels correlate with faster motor and cognitive decline, shorter survival, and more severe clinical progression, especially in atypical or aggressive PD phenotypes^24–26^. In our study, serum NfL levels were significantly elevated in the DM subtype compared to MMP and IM subtypes (*P* = 0.005, pairwise comparison), suggesting a greater burden of neurodegeneration. Further, we found that out of the examined biomarkers, serum NfL was a significant independent predictor of faster milestone attainment (*data not shown*), reinforcing its prognostic relevance. These findings support the emerging interpretation that NfL elevation may reflect an active axonal injury process that underpins the aggressive disease trajectory in the DM group. This aligns with prior findings by Pilotto et al., who identified that high NfL levels were associated with a more malignant PD phenotype and rapid motor progression over two years^24^.

Our findings align with and extend prior work by Fereshtehnejad et al.^14,27,28^, who first proposed the data-driven DM, IM, and MMP subtypes. This prospective validation in the PPMI cohort strengthens the clinical relevance of the data-driven subtyping framework. Further supporting the clinical significance of the DM subtype, Johansson et al.^29^ analyzed the two-year longitudinal data demonstrating that patients classified under the DM subtype exhibited significantly faster progression in both motor and non-motor symptoms compared to other subtypes. Their findings highlight the aggressive nature of the DM phenotype and underscore its potential as a critical target for stratified therapeutic interventions. In parallel, Hähnel et al.^30^ proposed another data-driven progression model using multi-cohort clustering of longitudinal symptom data. Their analysis identified distinct fast- and slow-progressing PD subtypes which may parallel the DM vs. MMP subtypes in Fereshtehnejad model^14,27,28^, a major shortcoming of their work is the lack of recommended or operational criteria to classify individual patients prospectively. This limits the clinical and translational applicability of their model.

On the other hand, our results challenge assumptions about pathological subtypes. The brain-first/body-first framework, based on Braak’s staging hypothesis^31^ suggests that body-first patients exhibit faster progression due to widespread synucleinopathy^32^. This is consistent with our results that body-first patients progressed faster than brain-first, however, their progression rates remained lower than those of DM, suggesting that pathological classifications may lack the granularity needed for trial stratification. A prior study by Xu et al*.*^19^. found faster disease progression in body-first PD compared to brain-first PD, however, they examined disease progression by the changes in MDS-UPDRS scores only^19^. In contrast, our study employed the 25 previously validated PPMI progression milestones^33^. The superiority of the DM subtype in predicting disease-progression may arise from the data-driven subtyping criteria itself. Since DM subtype is defined based on the scores of RBDSQ, MoCA, SCOPA-AUT, and MDS-UPDRS, it has more potential to capture individuals with impairment in multiple domains. However, in our study and others’^19^, the RBDSQ was used to define body-first/brain-first subtypes, which may not fully capture underlying neuropathology.

These findings have substantial implications for designing future disease-modification trials. Our simulations revealed that trials enrolling DM patients could achieve 80% statistical power with substantially smaller sample sizes (~50% fewer participants) and shorter durations compared to unstratified cohorts. In other words, for disease-modification trials aiming to slow the disease progression, prospective subtyping PD patients and enrollment of DM subtype will achieve greater statistical power and a higher likelihood of demonstrating treatment efficacy even with the same sample size and follow-up duration used in conventional trials of unstratified PD. This may be explained by the DM’s rapid progression, which enhances the sensitivity to detect treatment effects. Such stratification could lower trial costs, accelerate timelines, and minimize participant exposure to ineffective therapies. Furthermore, focusing on rapid progressors aligns with ethical obligations to prioritize patients most likely to benefit from early intervention. These advantages position data-driven subtyping as a promising strategy for overcoming historical challenges in PD disease-modification trials, where high heterogeneity has contributed to failures.

An important limitation of data-driven subtyping approaches, including the one employed here, is the population-relative nature of percentile-based cutoffs used to assign subtype membership. Because subtype boundaries are defined by the distribution of the study sample (the 75th percentile), the same individual patient could be classified into different subtypes depending on the composition and severity distribution of the cohort in which they are enrolled or depending on when in their disease course they are assessed. This has direct implications for the interpretation of subtype–biomarker associations: if a patient is categorized as DM in one cohort but as IM in another (with more severely affected participants) while their serum biomarker profile remains unchanged, the observed correlations between subtypes and biomarkers may partly reflect population-level statistical properties rather than stable, patient-intrinsic biological signatures. This does not invalidate the utility of data-driven subtypes for characterizing group-level heterogeneity or enriching clinical trial populations, but it does underscore the need for complementary approaches that anchor subtype definitions to absolute, biologically grounded thresholds (such as fixed biomarker cutoffs or normative reference values) rather than relying solely on within-cohort relative rankings. Future work should examine the stability of subtype assignments across independent cohorts and disease stages and explore whether integrating biological markers directly into subtype definitions could yield classifications that are more reproducible and patient intrinsic.

Currently, the neurobiological basis of PD subtypes remains unknown. For future studies, one interesting question is whether those patients with DM subtype have a higher burden of α-synuclein pathology and/or more aggressive microglial activation that contributes to the faster disease progression and the active neurodegenerative process (as reflected by the higher CSF and serum NfL levels). A recently made available preprint reported that Amplification parameters of the alpha-synuclein seed amplification assay on CSF were distinct between PD data-driven subtypes, suggesting that patients with more aggressive disease (those subtyped as DM) have shorter reaction time, which may reflect higher density of misfolded α-synuclein aggregates in the CSF sample^34^. Future advances in α-synuclein quantification methods and neuroimaging modalities would help better understanding of disease behavior in those patients. Lack of subtype stability has been criticized in the clinical subtyping model (TD, PIGD, and indeterminate), because disease subtypes may reflect different stages of the disease rather than distinct clinicopathological entities^21^. For this reason, future longitudinal studies tracking subtype stability over time are also needed, as PD heterogeneity may evolve with disease course. Finally, interventional trials should test whether subtype-specific therapies (e.g., targeting neuroinflammation in DM) yield superior outcomes compared to the “one-size-fits-all” approaches.

This study has several strengths: (1) the use of PPMI, a well-characterized large cohort of PD patients, (2) rigorous evaluation of disease progression across six functional domains instead of focusing on motor score only, (3) the integration of Cox proportional hazards regression and disease-modification trial size estimations further strengthen the clinical applicability of our findings. However, several limitations warrant consideration. First, the PPMI cohort comprises early-stage PD patients (Hoehn & Yahr I–II) without dementia, limiting generalizability to more advanced stages or those with significant cognitive impairment. Second, while milestone definitions were clinically meaningful, they may not fully reflect the biological process underlying the disease progression. Third, survival bias may exist if patients who dropped have different progression rates. We handled missing milestone assessments conservatively by assuming that if data were missing, the milestone was not yet reached. Finally, this is an exploratory analysis from the PPMI cohort, therefore, it requires validation in other independent populations, particularly those with diverse demographics and longer follow-up. Overall, the present findings suggest that data-driven subtyping, particularly the identification of the DM subtype, could optimize disease-modification clinical trials for PD. Given the rapid disease progression observed in this subtype, this approach is expected to increase the sensitivity of disease-modification trials to demonstrate benefits by enhancing statistical power, reducing trial costs, and accelerating the evaluation of disease-modifying therapies. As PD research advances toward precision medicine, the integration of multimodal biomarkers into subtyping algorithms will be crucial for elucidating the neurobiological basis of PD heterogeneity.

## Methods

### Study design

This study used data from the PPMI, a prospective, longitudinal, observational cohort study designed to identify biomarkers of PD progression. Ethical approval was obtained from institutional review boards at all participating PPMI sites, and written informed consent was provided by all participants. The current analysis was exempt from additional ethical review at Virginia Commonwealth University (VCU) as it involved secondary analysis of de-identified data. Reporting adhered to the STROBE (Strengthening the Reporting of Observational Studies in Epidemiology) guidelines for observational studies.

### Study participants

PPMI PD participants inclusion criteria were as follows: (1) diagnosis within the past two years, (2) Hoehn and Yahr stage I–II, and (3) presence of bradykinesia plus at least one additional motor symptom (resting tremor or rigidity). Individuals with a Montreal Cognitive Assessment (MoCA) score <21 were excluded. Data were extracted from the PPMI database on September 28, 2024, encompassing all eligible participants with a cohort definition of “*Parkinson’s Disease*”. We excluded patients in the Scans Without Evidence of Dopaminergic Deficit (SWEDD) cohort and prodromal cohort.

### Study assessments

Participants underwent standardized clinical and neuropsychological assessments, including global cognition measured by the MoCA, motor function evaluated using the Movement Disorder Society–Unified Parkinson’s Disease Rating Scale (MDS-UPDRS Parts I–III), autonomic symptoms assessed by the Scale for Outcomes in Parkinson’s Disease–Autonomic (SCOPA-AUT), olfaction measured by the University of Pennsylvania Smell Identification Test (UPSIT), and REM sleep behavior assessed using the REM Sleep Behavior Disorder Screening Questionnaire (RBDSQ).

Cerebrospinal fluid (CSF) biomarkers analyzed in this study included amyloid-beta 1–42 (Aβ₄₂), total tau (tTau), phosphorylated tau at threonine 181 (pTau), and neurofilament light chain (NfL). Serum NfL levels were also measured. To align with the subtyping time point, only biomarker measurements obtained at the baseline visit were included. Aβ₄₂, tTau, and pTau were quantified using Elecsys immunoassays (Roche), while CSF and serum NfL concentrations were measured using the Simoa platform (Quanterix).

### Subtyping Classifications

Three distinct subtyping frameworks were applied at baseline:

1. *Clinical Subtypes:* Participants were classified into TD, PIGD, or indeterminate categories based on the MDS-UPDRS following the recommended criteria^18^. The tremor score was derived from the sum of items 3.15–3.18 (resting tremor severity in limbs), while the PIGD score was calculated from items 2.12–2.13 (gait and postural instability) and 3.10–3.12 (axial motor features). Ratios of tremor to PIGD scores were computed, with thresholds of ≥1.15 defining TD, ≤0.9 defining PIGD, and values between 0.9 and 1.15 categorized as indeterminate.
2. *Pathological Subtypes:* We classified the body-first and proposed brain-first subtypes based on RBD status, consistent with the framework of Borghammer and Van Den Berge^35^ and its clinical operationalization using RBDSQ scores as applied by Horsager et al.^32^ and Xu et al.^19^. Given the lack of complete multimodal imaging battery in the PPMI, brain-first and body-first subtypes were defined similar to Xu et al.^19^ using the RBDSQ. Participants with RBDSQ scores ≤3, indicating minimal REM sleep dysfunction, were classified as brain-first. Those with scores ≥6, reflecting prominent REM sleep features, were classified as body-first. Individuals scoring 4–5 were excluded to ensure clear distinction between subtypes.
3. *Data-Driven Subtypes:* Subtypes (DM, IM, and MMP) were assigned using criteria established by Fereshtehnejad et al.^14^. Composite motor scores were computed as the average of z-standardized MDS-UPDRS Part II total, MDS-UPDRS Part III total, and PIGD ratio scores. The PIGD ratio was calculated as the sum of three postural instability/gait items (NP3GAIT, NP3FRZGT, NP3PSTBL) divided by the sum of ten tremor items from Part III, following the original algorithm. Non-motor composite scores included SCOPA-AUT for autonomic dysfunction, RBDSQ for REM sleep behavior disorder, and MoCA for cognition. Using data from the full cohort (*n* = 2019 PD patients), we calculated the 75th percentiles for composite motor, SCOPA-AUT, and RBDSQ scores; for MoCA, we used the 25th percentile to account for its inverse scoring direction. Patients were classified as DM if their composite motor score exceeded the 75th percentile and at least one non-motor domain also exceeded its threshold, or if all three non-motor domains exceeded their thresholds; MMP if all scores were below their respective thresholds; and IM otherwise. Of the total cohort, 1431 patients had complete data for all six variables required for classification.

### Progression milestones tracking

Progression milestones were assessed longitudinally across all available follow-up visits in the PPMI cohort as described before^33^. A total of 25 clinically meaningful milestones were defined across six domains: walking and balance, motor complications, cognition, autonomic dysfunction, functional dependence, and activities of daily living. Milestones were evaluated during all available scheduled visits up to 10 years. Participants were considered milestone-positive once a participant meets any one of the 25 milestones. Once a milestone was attained, participants were classified as having reached that milestone, and progression rates were analyzed both overall and by domain. Missing data were conservatively handled by assuming that milestone criteria were not met when assessments were unavailable. Further analysis was conducted where we excluded baseline-positive participants since they cannot contribute to time-to-event estimation.

### Study size and power calculation

This study analyzed all eligible participants from the PPMI cohort, yielding a total sample size of 2019 patients. No formal priori sample size calculation was performed given the secondary nature of the analysis. However, estimations indicated that the available sample provided sufficient statistical power. For comparisons of milestone attainment rates across subtypes, the study had >80% power to detect absolute differences of 10–15% between groups at a two-tailed significance level of 0.05, corresponding to a medium-to-large effect size (Cohen’s *h* ≈ 0.4–0.6). In Cox proportional hazards regression analyses, the sample size provided >80% power to detecthazard ratios (HRs) of 1.5 or greater, assuming an event rate of approximately 30% and an α = 0.05. Given observed HR estimates ranging from 1.36 to 2.06 in key comparisons, and the substantial number of events recorded, the study was adequately powered to detect clinically meaningful differences across subtypes.

### Statistical analysis

Continuous variables were summarized as median and interquartile range (IQR), while categorical variables were presented as frequencies and proportions. For comparisons between two groups (pathological subtypes: brain-first vs. body-first), the Wilcoxon rank-sum test was used. For comparisons across three groups (clinical subtypes: TD, PIGD, indeterminate; and data-driven subtypes: DM, IM, MMP), the Kruskal-Wallis test was applied for continuous variables. Pearson chi-square tests were used to assess differences in categorical variables. Cox proportional hazards regression models, using the Efron method for tied events, were employed to estimate hazard ratios (HRs) for milestone attainment across subtypes, adjusting for age at enrollment. Statistical significance was set at a two-tailed *P* < 0.05. All analyses were conducted using Jamovi version 2.3.28 (The Jamovi Project), Stata/MP 18.0 (StataCorp, College Station, TX, USA), and R version 4.5.0 (R Foundation for Statistical Computing, Vienna, Austria) within the RStudio integrated development environment (version 2024.12.1 + 563; Posit Software, PBC, 2025).

### Hypothesized disease-modifying trial sample size estimations

We estimated the minimum sample sizes required for disease-modifying trials in Parkinson’s disease using observed survival probabilities and a hypothesized treatment effect. Patients who had reached any progression milestone at baseline were excluded to ensure that only incident milestones were considered. Time-to-event data were structured using Stata (*stset*), with event status coded as failure. Survival probabilities at 1–10 years were extracted for each subtype using the Kaplan–Meier method (*sts list by subtype*). Progression event probabilities were calculated as (1 − survival probability). Assuming a 20% relative reduction in milestone progression (HR = 0.80), we applied standard formulas for time-to-event sample size estimation with 80% power and a two-sided alpha of 0.05. This effect size (20% efficacy) is supported by a recent modeling study by Ribba et al., which simulated PD progression and evaluated treatment effects ranging from 20 to 50% slowing of decline using longitudinal data from PPMI and PASADENA trial^36^. To address the potential confounding effect of age on subtype-specific progression rates, we performed a sensitivity analysis using 1:1 nearest-neighbor matching on enrollment age with a fixed caliper of 2 years. For each pairwise comparison, patients in the smaller group were matched (in random order) to the nearest unmatched patient in the larger group. Each control was used at most once. Cox proportional hazards regression was then fitted on the matched sample, with the subtype indicator and enrollment age as covariates. Balance was verified using the Kolmogorov-Smirnov test and the Wilcoxon rank-sum test. All estimations were implemented in Python 3.10 (Google Colab), using survival probabilities derived from Stata/MP 18.0.

## Supplementary information

Supplementary information

## Data Availability

The datasets analyzed during the current study are publicly available from the Michael J. Fox’s PPMI database: http://PPMI-info.org.

## Code availability

The statistical analysis files: Stata script files (.do), Jamovi files (.omv), and the final dataset (.csv) are available from the corresponding author on reasonable request.

## References

[1] Dickson, D. W. Neuropathology of Parkinson disease. *Parkinsonism Relat. Disord.* **46**, S30–S33 (2018). 10.1016/j.parkreldis.2017.07.033 PMC5718208 28780180

[2] Wong, Y. et al. Neuronal vulnerability in Parkinson disease and putative therapeutics: should the focus be on axonal and synaptic terminals? *Mov. Disord. J. Mov. Disord. Soc.* **34**, 1406–1422 (2019). 10.1002/mds.27823 PMC6879792 31483900

[3] He, S., Zhong, S., Liu, G. & Yang, J. Alpha-synuclein: the interplay of pathology, neuroinflammation, and environmental factors in Parkinson’s disease. *Neurodegener. Dis.* **20**, 55–64 (2020). 10.1159/000511083 33465773

[4] Cuenca, L. et al. Parkinson’s disease: a short story of 200 years. *Histol. Histopathol.* **34**, 573–591 (2019). 10.14670/HH-18-073 30540129

[5] Marras, C. et al. Prevalence of Parkinson’s disease across North America. *NPJ Park Dis.* **4**, 21 (2018). 10.1038/s41531-018-0058-0 PMC6039505 30003140

[6] Negida A, et al. Epidemiology and risk factors of Parkinson’s disease. in *Essential Guide to Neurodegenerative Disorders* (eds Mohamed WMY) (Academic Press, 2025).

[7] Chaudhuri, K. R. & Naidu, Y. Early Parkinson’s disease and non-motor issues. *J. Neurol.* **255**, 33–38 (2008). 10.1007/s00415-008-5006-1 18787880

[8] Akbar, U., D’Abreu, A. & Friedman, J. H. Nonmotor symptoms in Parkinson’s disease. *Semin. Neurol.* **37**, 158–166 (2017). 10.1055/s-0037-1602424 28511256

[9] Moustafa, A. A. et al. Motor symptoms in Parkinson’s disease: a unified framework. *Neurosci. Biobehav. Rev.* **68**, 727–740 (2016). 10.1016/j.neubiorev.2016.07.010 27422450

[10] Stocchi, F., Bravi, D., Emmi, A. & Antonini, A. Parkinson disease therapy: current strategies and future research priorities. *Nat. Rev. Neurol.* **20**, 695–707 (2024). 10.1038/s41582-024-01034-x 39496848

[11] Suhas, M. & Kadiri, S.K. Parkinson’s disease: from bench to bedside-advancements in diagnosis and therapeutics using pharmacogenomic approach. *Curr Gene Ther*. 10.2174/0115665232342211250207064205 (2025). 39945257

[12] Adams, C. et al. Updated Parkinson’s disease motor subtypes classification and correlation to cerebrospinal homovanillic acid and 5-hydroxyindoleacetic acid levels. *Clin. Park Relat. Disord.* **8**, 100187. 10.1016/j.prdoa.2023.100187 (2023). PMC9922918 36793590

[13] Belvisi, D. et al. The pathophysiological correlates of Parkinson’s disease clinical subtypes. *Mov. Disord. J. Mov. Disord. Soc.* **36**, 370–379 (2021). 10.1002/mds.28321 33037859

[14] Fereshtehnejad, S.-M., Zeighami, Y., Dagher, A. & Postuma, R. B. Clinical criteria for subtyping Parkinson’s disease: biomarkers and longitudinal progression. *Brain J. Neurol.* **140**, 1959–1976 (2017). 10.1093/brain/awx118 28549077

[15] Mestre, T. A. et al. Parkinson’s disease subtypes: critical appraisal and recommendations. *J. Park Dis.* **11**, 395–404 (2021). 10.3233/JPD-202472 PMC8150501 33682731

[16] Bohnen, N. I. & Postuma, R. B. Body-first versus brain-first biological subtyping of Parkinson’s disease. *Brain J. Neurol.* **143**, 2871–2873 (2020). 10.1093/brain/awaa293 PMC7821233 33103732

[17] Prime, M. et al. Differentiating Parkinson disease subtypes using clinical balance measures. *J. Neurol. Phys. Ther.* **44**, 34–41 (2020). 10.1097/NPT.0000000000000297 PMC6991154 31834219

[18] Stebbins, G. T. et al. How to identify tremor dominant and postural instability/gait difficulty groups with the movement disorder society unified Parkinson’s disease rating scale: comparison with the unified Parkinson’s disease rating scale. *Mov. Disord. J. Mov. Disord. Soc.* **28**, 668–670 (2013). 10.1002/mds.25383 23408503

[19] Xu, Z. et al. Disease progression in proposed brain-first and body-first Parkinson’s disease subtypes. *NPJ Park Dis.* **10**, 111 (2024). 10.1038/s41531-024-00730-1 PMC11150376 38834646

[20] Berg, D. et al. Time to Redefine PD? Introductory statement of the MDS task force on the definition of Parkinson’s disease. *Mov. Disord.* **29**, 454–462 (2014). 10.1002/mds.25844 PMC4204150 24619848

[21] von Coelln, R. et al. The inconsistency and instability of Parkinson’s disease motor subtypes. *Parkinsonism Relat. Disord.* **88**, 13–18 (2021). 10.1016/j.parkreldis.2021.05.016 34091412

[22] Zuo, L.-J. et al. Phenotype of postural instability/gait difficulty in Parkinson disease: relevance to cognitive impairment and mechanism relating pathological proteins and neurotransmitters. *Sci. Rep.* **7**, 44872 (2017). 10.1038/srep44872 PMC5362957 28332604

[23] Kang, J.-H. et al. Association of cerebrospinal fluid β-Amyloid 1-42, T-tau, P-tau181, and α-synuclein levels with clinical features of drug-naive patients with early Parkinson disease. *JAMA Neurol.* **70**, 1277–1287 (2013). 10.1001/jamaneurol.2013.3861 PMC4034348 23979011

[24] Pilotto, A. et al. Plasma NfL, clinical subtypes and motor progression in Parkinson’s disease. *Parkinsonism Relat. Disord.* **87**, 41–47 (2021). 10.1016/j.parkreldis.2021.04.016 33964785

[25] Lin, C-H. et al. Blood NfL: a biomarker for disease severity and progression in Parkinson disease. Neurology **93**. 10.1212/wnl.0000000000008088 (2019). 31420461

[26] Bäckström, D. et al. NfL as a biomarker for neurodegeneration and survival in Parkinson disease. *Neurology* **95**, e827–e838 (2020). 10.1212/WNL.0000000000010084 PMC7605503 32680941

[27] Fereshtehnejad, S.-M. & Postuma, R. B. Subtypes of Parkinson’s Disease: what do they tell us about disease progression?. *Curr. Neurol. Neurosci. Rep.* **17**, 34 (2017). 10.1007/s11910-017-0738-x 28324303

[28] Fereshtehnejad, S.-M. et al. New clinical subtypes of Parkinson disease and their longitudinal progression: a prospective cohort comparison with other phenotypes. *JAMA Neurol.* **72**, 863–873 (2015). 10.1001/jamaneurol.2015.0703 26076039

[29] Johansson, M. E., van Lier, N. M., Kessels, R. P. C., Bloem, B. R. & Helmich, R. C. Two-year clinical progression in focal and diffuse subtypes of Parkinson’s disease. *NPJ Park Dis.* **9**, 29 (2023). 10.1038/s41531-023-00466-4 PMC9937525 36806285

[30] Hähnel, T. et al. Progression subtypes in Parkinson’s disease identified by a data-driven multi cohort analysis. *NPJ Park Dis.* **10**, 95 (2024). 10.1038/s41531-024-00712-3 PMC11066039 38698004

[31] Braak, H. et al. Staging of brain pathology related to sporadic Parkinson’s disease. *Neurobiol. Aging* **24**, 197–211 (2003). 10.1016/s0197-4580(02)00065-9 12498954

[32] Horsager, J. et al. Brain-first versus body-first Parkinson’s disease: a multimodal imaging case-control study. *Brain* **143**, 3077–3088 (2020). 10.1093/brain/awaa238 32830221

[33] Brumm, M. C. et al. Parkinson’s progression markers initiative: a milestone-based strategy to monitor parkinson’s disease progression. *J. Park Dis.* **13**, 899–916 (2023). 10.3233/JPD-223433 PMC10578214 37458046

[34] Grillo P, Riboldi GM, Pisani A, Kang UJ, Fereshtehnejad SM. Faster reaction times of CSF alpha-synuclein seed amplification assay predict the diffuse malignant subtype of Parkinson's disease at 10-year follow-up. J Parkinsons Dis. **16**, 233–245 (2026). 10.1177/1877718X261417468 PMC13347583 41610263

[35] Borghammer, P. & Van Den Berge, N. Brain-first versus gut-first Parkinson’s Disease: a hypothesis. *J. Park Dis.* **9**, S281–S295 (2019). 10.3233/JPD-191721 PMC6839496 31498132

[36] Ribba, B. et al. Modeling of Parkinson’s disease progression and implications for detection of disease modification in treatment trials. *J. Park Dis.* **14**, 1225–1235 (2024). 10.3233/JPD-230446 PMC11380229 39058452

