# Supplementary material for: Comparative analysis of progression milestones across Parkinson’s disease clinical, pathological, and data-driven subtypes: a 10-year follow-up
Source: NPJ Parkinsons Dis. 2026 Jun 12;12:239. doi: 10.1038/s41531-026-01430-8 (PMC13616926; doi:10.1038/s41531-026-01430-8)
Supplement: Supplementary file 1 [file 41531_2026_1430_MOESM1_ESM.docx]

# Supplementary Table: Age-Matched Sensitivity Analysis

## Table S1. Cox Proportional Hazards Regression on Age-Matched Samples

| **Framework** | **Comparison** | **N/group** | **HR** | **95% CI** | **P (Cox)** | **P (log-rank)** |
| --- | --- | --- | --- | --- | --- | --- |
| **Clinical** | PIGD vs TD | 327 | **2.08** | 1.67–2.60 | **<0.001** | <0.001 |
|  | Indeterminate vs TD | 110 | 1.39 | 0.92–2.09 | 0.115 | 0.079 |
| **Pathological** | Body-first vs Brain-first | 374 | **2.06** | 1.66–2.56 | **<0.001** | <0.001 |
| **Data-driven** | IM vs MMP | 446 | **1.85** | 1.50–2.29 | **<0.001** | <0.001 |
|  | DM vs MMP | 210 | **4.94** | 3.58–6.81 | **<0.001** | <0.001 |
|  |  |  |  |  |  |  |

*Abbreviations: HR, hazard ratio; CI, confidence interval; TD, tremor-dominant; PIGD, postural instability and gait difficulty; MMP, mild motor-predominant; IM, intermediate; DM, diffuse malignant. Bold indicates P < 0.05. Cox models adjusted for enrollment age.*

## Table S2. Age Balance Verification After Matching

| **Comparison** | **Treated Age, mean (SD)** | **Control Age, mean (SD)** | **Mean Difference** | **KS test P** | **Rank-sum P** | **Balance** |
| --- | --- | --- | --- | --- | --- | --- |
| PIGD vs TD | 63.3 (10.0) | 63.3 (10.0) | 0.0 | 1.000 | 0.995 | **Yes** |
| Indeterminate vs TD | 61.9 (9.8) | 61.8 (9.8) | 0.1 | 1.000 | 0.989 | **Yes** |
| Body-first vs Brain-first | 63.4 (9.5) | 63.4 (9.5) | 0.0 | 1.000 | 0.997 | **Yes** |
| IM vs MMP | 63.2 (9.1) | 63.1 (9.1) | 0.1 | 0.997 | 0.964 | **Yes** |
| DM vs MMP | 64.4 (9.7) | 64.3 (9.7) | 0.1 | 1.000 | 0.997 | **Yes** |
|  |  |  |  |  |  |  |

*Note: All KS test and rank-sum P-values are non-significant, confirming adequate age balance across all matched comparisons.*

## Table S3. Comparison of Unmatched vs Age-Matched Results

| **Comparison** | **Unmatched (Original)** | | | **Age-Matched** | | |
| --- | --- | --- | --- | --- | --- | --- |
|  | **HR** | **95% CI** | **P** | **HR** | **95% CI** | **P** |
| PIGD vs TD | 1.63 | 1.37–1.94 | <0.001 | **2.08** | 1.67–2.60 | **<0.001** |
| Indeterminate vs TD | 0.88 | 0.65–1.19 | 0.409 | **1.39** | 0.92–2.09 | **0.115** |
| Body-first vs Brain-first | 2.01 | 1.66–2.43 | <0.001 | **2.06** | 1.66–2.56 | **<0.001** |
| IM vs MMP | 1.91 | 1.57–2.32 | <0.001 | **1.85** | 1.50–2.29 | **<0.001** |
| DM vs MMP | 4.16 | 3.29–5.27 | <0.001 | **4.94** | 3.58–6.81 | **<0.001** |
|  |  |  |  |  |  |  |

*Note: Hazard ratios remain significant and directionally consistent after age matching for all comparisons except Indeterminate vs TD (which was non-significant in both analyses). This confirms that the observed subtype differences in progression are not driven by age confounding.*

## Interpretation

After 1:1 age matching with a 2-year caliper, subtype-specific differences in progression to disability milestones remained statistically significant and consistent with the primary analysis. PIGD patients progressed faster than TD patients (HR 2.08, 95% CI 1.67–2.60, P < 0.001), Body-first patients progressed faster than Brain-first patients (HR 2.06, 95% CI 1.66–2.56, P < 0.001), and both IM (HR 1.85, 95% CI 1.50–2.29, P < 0.001) and DM (HR 4.94, 95% CI 3.58–6.81, P < 0.001) subtypes progressed faster than MMP. The Indeterminate vs TD comparison remained non-significant (HR 1.39, 95% CI 0.92–2.09, P = 0.115), consistent with the primary analysis. These findings confirm that the observed differences in disease progression across all three subtyping frameworks are not attributable to age confounding.
